# Boolean Networks as Modeling Framework

**DOI:** 10.3389/fpls.2012.00178

**Published:** 2012-08-15

**Authors:** Florian Greil

**Affiliations:** ^1^Lehrstuhl für Bioinformatik, Universität LeipzigLeipzig, Germany; ^2^Climate Science Division, Observational Oceanography, Alfred-Wegener-Institut für Polar- und MeeresforschungBremerhaven, Germany

**Keywords:** boolean networks, dynamics on networks, complex systems, relevant component

## Abstract

In a network, the components of a given system are represented as nodes, the interactions are abstracted as links between the nodes. Boolean networks refer to a class of dynamics on networks, in fact it is the simplest possible dynamics where each node has a value 0 or 1. This allows to investigate extensively the dynamics both analytically and by numerical experiments. The present article focuses on the theoretical concept of relevant components and their immediate application in plant biology. References for more in-depth treatment of the mathematical details are also given.

Networks are used to model various complex systems whose units interact in an intricate manner. The units are represented by nodes and the interactions by links. Topology studies the static structure of a network while the dynamics *on* a network describes what happens on a given realization of a network, i.e., on a fixed topology. This article focuses on the *simplest dynamics on a network*, each node *i* having a Boolean value σ*_i_ *∈ {0, 1} which may change in time while the topology remains fixed.

Although Boolean models represent a strong simplification of reality, for several cases they were shown to correctly capture the essential dynamics such as the correct pattern of expressed and suppressed genes, see (Albert and Othmer, 2003; Li et al., 2004).

## The *N*-*K*-Model

**Figure 1:**
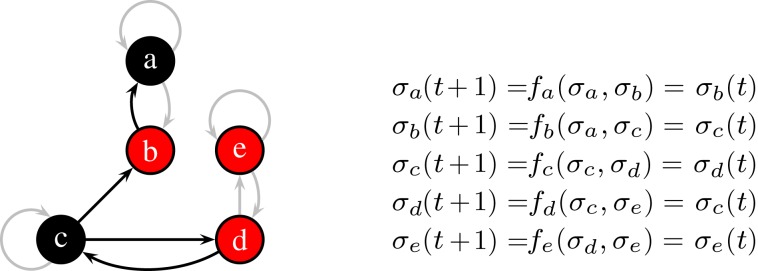
**At the current time step, node *a* and *c* are off (0, black), the others are on (1, red)**. The functions *f_a_…*e** with *K *= 2 effectively depend only on one input, non-used links are grayed-out. Here, all functions describe a simple “copy”-operation, e.g., *f_a_* says that node *a* will take the value σ*_b_* at the next time step.

Kauffman (1969) introduced a Boolean network he called *N*-*K*-model. In this model each of the *N* nodes have exactly *K* incoming links. Topologically, it is a directed graph with *N* nodes and *N*·*K* random links in between. The dynamics is incorporated by a Boolean function *f_i_* at each node *i* which describes how the value of a given node changes with time. In spite of its simplicity, the model has not been understood analytically until the 2000s. Figure 1 shows an *N*-*K*-model with *N *= 5 and *K *= 2.

The dynamics, i.e., how the nodes blink, can be quantified by the statistics of the *attractors*. An attractor is a series of repeating system states which is reached after passing through a number of transient system states. The set of all possible patterns of node values forms the *state space* of a network, consisting of 2*^N^* states. It is important to understand the difference between node space and state space. For gene regulation, node space contains all genes while state space comprises all (mathematically) possible expression patterns. In node space, the links symbolize how genes influence each other. In state space, the links between different expression patterns stand for predecessor-successor relationships.

**Figure 2:**
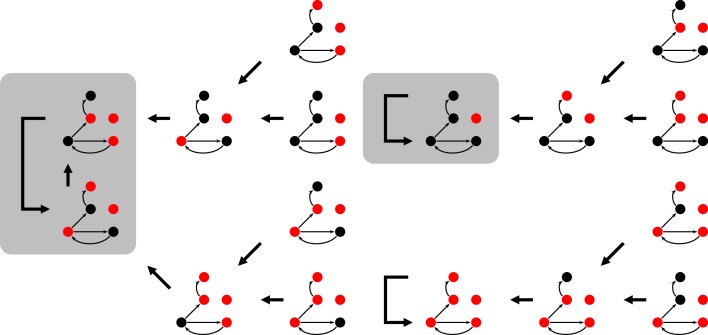
**Part of the state space for Figure 1 under synchronous updating**. Each snapshots is a system state, the thick arrows indicate which state follows in time. Depending on the initial state, the system ends up on one of the attractors (shaded rectangles). For the 16 (of 2^5^) states shown here, it can either be a 2-cycle or one of the two fixed points.

The state space of *N*-*K* networks is finite (even if extremely large) and the dynamics is deterministic. These two ingredients guarantee that a time series of repeating states will eventually end up on an *attractor*, like those depicted by the gray boxes in Figure 2.

The mean number and length of attractors in critical networks has been the subject of a number of publications (see Appendix). Here they should serve as an example system to demonstrate the method of relevant components. Kauffman expected the attractor length to scale with the square-root of the number of nodes, this fit his idea that attractors correspond to cell-types and nodes correspond to genes. However, it turned out, that some of the numerical results were actually artifacts of the limited computer power back then. Some decades later, Samuelsson and Troein (2003) contributed an elegant but elaborate proof which ended the discussion.

## Dynamical Components

The so-called *relevant components* are key ingredients for calculating the number and length of attractors of a Boolean network. The main idea is that there are three types of nodes:

1. *Frozen nodes* (like node *e* in Figure 1 which is always red) stop blinking after some time and are afterward no longer important for the dynamics.
2. *Irrelevant blinking nodes* (node *a* and *b* in Figure 1) have dynamics completely determined by other nodes. They are arranged as a subset of nodes without any cycles. Nodes which do not influence any other node can be cut off when searching for the attractor. After such a pruning step there are possibly new nodes which do not influence any other node and one can repeat the pruning step as long as there are nodes with no outgoing links. We will end up with cutting all outgoing trees of nodes whose dynamics is irrelevant for the length of the attractor.
3. Finally, only *relevant nodes* (nodes *c* and *d* in Figure 1) are left, each influences at least one other relevant node.

The relevant nodes form relevant components. It has been shown that there are of the order of log *N* such components. Furthermore, it is known that most components are simple loops (like nodes *c* and *d* in Figure 1). Usually only the largest component is more complex, e.g., having an extra link within. If (i) the distribution of the components is known and (ii) the dynamics of each of them is understood, the overall attractor length of the system can be calculated. Simple combinatorics say that the least common multiple of the individual components’ attractor lengths give the overall attractor length of a given network realization, see, e.g., Reviews (Aldana et al., 2003; Drossel, 2008).

Coexpressed genes might be blinking, but that does not necessarily mean that they are relevant. If one uses an algorithm to construct a network topology based on coexpression it cannot be decided yet. Once when the regulatory functions are identified, the method of relevant components can be applied and this possibly leads to more insight into the dynamical role of each node.

## Modifying the Updating Scheme

Up to now it was quietly assumed that all nodes of the networks are updated synchronously at discrete time steps. The parallel update of all nodes at the same time is handy for computer experiments, but on biological scales time is continuous and this assumption of synchronous update usually does not hold. To account for this flaw, different asynchronous updating schemes have been introduced, the extreme is a fully asynchronous version where the value of a given node is changed at random times (but still according to a fixed Boolean function). The approach of the relevant components can still be applied since it is independent of the updating scheme. Again, the knowledge about (i) the statistics of the relevant components and (ii) the individual dynamics is put together and a conclusion for the overall dynamics can be drawn.

For asynchronously and stochastically updated *N*-*K*-models one finds the original square-root behavior for the scaling of the attractor length with the network size (Greil and Drossel, 2005). The main reason for this result is that the number of repeating states per component becomes smaller while the distribution remains the same.

## Modifying the Network Topology

In the previous section the distribution of relevant components remained unchanged while the components’ dynamics was modified, now it is vice versa. Assuming synchronous dynamics, the effect of topological changes can be studied. As there are no living systems where *all* genes are regulated by *exactly* two other genes, one can have look at a scale-free in-degree distribution, i.e., the fraction *P*(*K*) of nodes having *K* in-links scales as *P*(*K*) ∼ *K*^−γ^ (with typically 2 < γ < 3). Such scale-free networks are found to be abundant in nature (Albert and Barabási, 2002).

It is possible to derive a formula for the number of non-frozen nodes (Drossel and Greil, 2009) which helps to setup the distribution of relevant components and eventually leads to mathematical statements about the dynamics.

For biology, applying Boolean models to experimentally determined topologies is more interesting. The Boolean toolbox has been successfully applied to various gene regulation networks of different species (Wang and Albert, 2009). Let us conclude with showing how the gene regulation network of *Arabidopsis thaliana*’s leaf epidermis gene regulation can be translated into Boolean language and the state space can be simplified. This system is a model experimental system in plant biology (Grebe, 2012). Starting point for our analysis is the dynamic model by Benítez et al. (2008) who integrated experimental data into a model which allowed to recover spatial cell patterns on leaf epidermis. The network is given as mixture of node and state space (see Appendix) which makes it more complicated to understand the dynamics. For the purpose of the current review it is not so important to which biological state a given state of the network actually correspond, but to see that the abstract method of relevant components is indeed applicable to biological systems.

**Figure 3:**
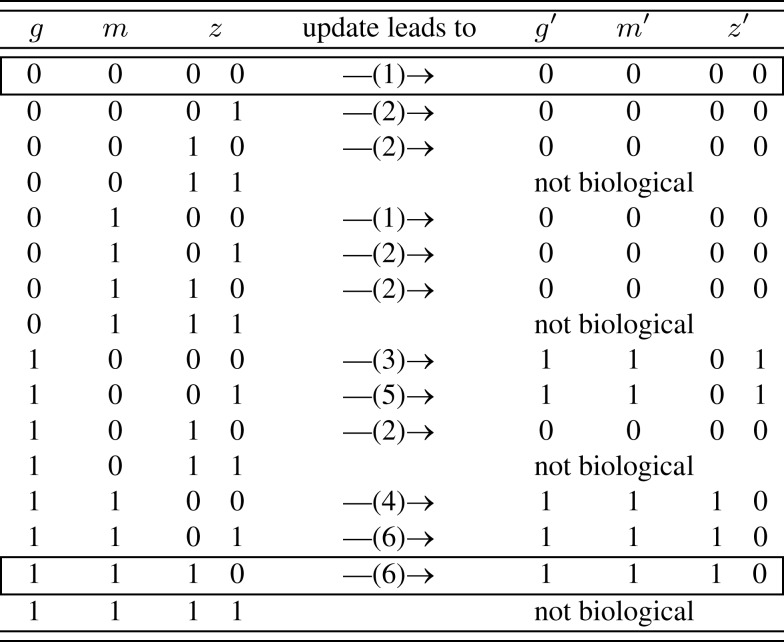
**Simplified state space of the leaf epidermis gene regulatory network where the variables *g*, *m*, *z* have an indirect biological meaning, see** Appendix. The number in brackets gives the corresponding row of Table A4 in Appendix. The state *z* is represented by two internal Boolean variables, as a side effect there are states without biological counter-part. The framed rows are the two fixed points.

Ideally, the physical network is given in *node space* (e.g., Figure 1) and *state space* (e.g., Figure 2) can be deduced from that. Untangling the complicated if-then-clauses of the leaf epidermis gene regulation leads to the state space given in Figure 3 which is less intuitive than Figure 2, but again carries the information on which network state is followed by a given expression pattern. Some of the genes have been discarded to simplify the analysis of the state space. The discarded nodes were actually relevant in the above definition since they are part of at least one connection loop. However, we can still leave them out since the values of the nodes are again entirely determined by the remaining ones. The applied procedure can roughly be visualized as “chain shrinking,” i.e., a node *C* that simply copies the value (“on”/“off”) of its predecessor *P* and hands this on to another node *A* can be discarded and *P* is directly wired to *A* (the Boolean function of node *A* is adjusted if necessary).

A closer look at the state space reveals that the only attractors are two fixed points, namely (*g*, *m*, *z*) ∈ {(0, 0, 00), (1, 1, 10)}. Biologically, (0, 0, 00) corresponds to all genes unexpressed. On the other hand, (1, 1, 10) means that *GL1* is expressed, while *GL3*, *CPC*, *ETC*, and *TRY* are highly expressed. One may speculate that the attractor (0, 0, 00) is more robust than (1, 1, 10) since its *basin of attraction* is larger: in 5/12 of the cases this attractor is reached assuming that all expression patterns are equal probable.

Putting everything together, the 3^7^ different possible expression states in *A. thaliana*’s leaf epidermis network (Benítez et al., 2008) have been reduced to only 12 states which less than 1% of the original state space. This simplification allowed to draw a conclusion simply from looking at the network and its state space – a clear benefit of the Boolean networks modeling framework.

## Conflict of Interest Statement

The author declares that the research was conducted in the absence of any commercial or financial relationships that could be construed as a potential conflict of interest.

## Figures and Tables

**Figure A1:**
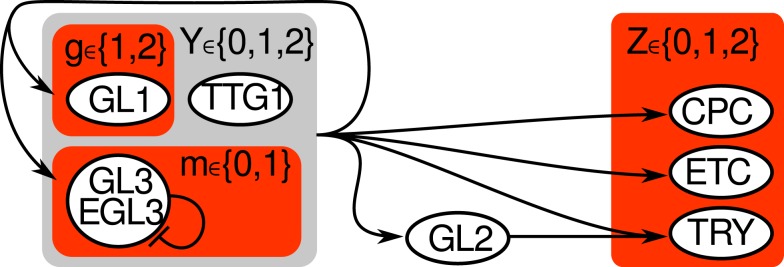
**Hybrid sketch of *Arabidopsis thaliana*’s leaf epidermis regulation before and after the simplification**. All nodes from the original variant (Benítez et al., 2008) are labeled by 3 or 4 letters. The simplified system consists of three nodes *g*, *m*, *z* only, highlighted by red shaded rectangles. The auxillary variable *Y* (light gray shaded) is not required any more, while *Z *∈ {0, 1, 2} is transformed into two Boolean nodes *z *∈ {00, 01, 10, 11}.

## Appendix

### Critical random boolean networks

In the manuscript critical random Boolean networks have been chosen as example system to present the concept of relevant components. The term “critical” needs more explanation.

In general, the dynamics of a Boolean network can be classified into two phases. In the *frozen* phase, all nodes apart from a small number have a constant value after a certain transient time (the number of updates until an attractor is reached). If the value of one node is changed in the stationary state, the perturbation propagates during one time step to less than one other node on average. In the *chaotic* phase, attractors are long, and a non-vanishing proportion of all nodes keep changing their value even after long times. A change of the value of a node affects on average more than a single node.

The focus usually lays at the border between the two phases, on so-called *critical* networks, since they combine *stability* against node-flips and *adaptability* in terms reasonable long transient times. Both features mimic biology where not every fluctuating environment should cause a change of the regulation, but if it does, it should happen reasonably fast.

As mentioned in the main text, the mean number and length of attractors in critical networks have been the subject of a number of publications, see Table A1 in Appendix. The discussion was put to an end by mathematical proofs.

**Table A1:** **Different results for the mean number 〈*v*〉 and the mean length 〈*A*〉 of attractors in the course of time**.

Kauffman (1969)	1969	〈 ν 〉, 〈A〉 ∼ $\sqrt{N}$
Bastolla/Parisi, Physica D 115	1998	〈*v*〉, 〈A〉 > *N ^x^* ∀*_x_*
Bilke/Sjunnesson, Phys. Rev. E 65	2002	〈*v*〉 ∼ *N*
Socolar/Kauffman, PRL 90	2003	〈*v*〉 ∼ *N ^x^*, *x *> 1
Samuelsson and Troein (2003)	2003	〈*v*〉 >*N ^x^* ∀*x*
Mihaljev/Drossel, Phys. Rev. E 74	2005	〈*v*〉, 〈A〉 > *N ^x^* ∀*x*

**N* is the number of nodes in the network. The last two papers are fully analytical*.

### Simplifying *Arabidopsis*’ leaf epidermis network

The purpose of this section is to illustrate which nodes of the original *A. thaliana*’s leaf epidermis discrete network model have been clustered (*CPC*, *ETC*, *TRY*) or left out (*GL2*, *TTG1*) and for what reason, see Figure A1 in Appendix for an overview.

**Table A2:** ***X* indicates that, given the entrances of the rest of the table, the node can take any value**.

*GL*1	*GL*3	E*GL*3	*TTG*1	*Y*
0	X	X	X	0
X	*GL*3 + *EGL*3 = 0		X	0
X	*GL*3 + *EGL*3 < 3		0	0
1	2	2	1, 2	2
2	2	X	1, 2	2
2	1	2	1, 2	2
Else				1

*The involved genes are *GLabra* and *TransparentTestaGlabra*. The table is identical to the original one in the Appendix of Benítez et al. (2008)*.

**Table A3:** **Table for *Z*, cited from Benítez et al. (2008)**.

*TRY*	*CPC*	*ETC*	*Z*
0	*CPC *+ *ETC *< 2		0
0	2	0	0
2	*CPC *+ *ETC *> 0		2
1	2	*X*	2
Else			1

*The rules hold for *CaPriCe*, *TRiptYchon* and the *EnhancerOfTryAndCpc1**.

**Table A4:** **The overall dynamics can be formulated in terms of *Y* and *Z***.

*Y*	*Z*	*GL*1	*(E)GL*3	*GL*2	*TTG*1	*ETC*	*CPC*
0	0	1	0	0	1	0	0
*Y *< *Z*		1	0	0	1	0	0
1	0	2	1	1	1	1	1
2	0	2	1	2	1	2	2
1	1	2	1	1	1	1	1
2	1,2	2	1	2	1	2	2

*In contrast to Benítez et al. (2008), the columns for *EGL3* and *GL3* are merged*.

The genes within the set *Z* are inhibitors preventing the expression of *GL2*, a gene that is only expressed in the leaves of the plant. This produces proteins which form protein complex that up-regulates *GL2*.

After evaluating Tables A2 and A3 in Appendix, we have values for *Y* and *Z* with which we go into Table A4 in Appendix to determine the succeeding system state. All this is performed within a single update step of the whole network.

These original conditions can be simplified by the following steps:

1. Leaving out *EGL3* is a reasonable from a dynamical perspective since it is nearly entirely determined by *GL3*.
2. Since *GL2* is a simple copy-node, we can cut out the node such that *TRY* receives its input from *Y* directly. This *pruning-procedure* is common to reduce the state space’s size.
3. Since *CPC*, *ETC*, and *TRY* always change their state together, we only take into account the state of the activator complex *Z*.

In the main text, we abbreviated *GL3* as *m* (for main), *GL1* as *g* and *z* is the Boolean representation of *Z*. Note that we changed the set of values from *GL1* ∈ {1, 2} to *g *∈ {0, 1}.
